# Influence of temperature, pH and simulated biological solutions on swelling and structural properties of biomineralized (CaCO_3_) PVP–CMC hydrogel

**DOI:** 10.1007/s40204-015-0043-1

**Published:** 2015-11-02

**Authors:** Rushita Shah, Nabanita Saha, Petr Saha

**Affiliations:** Centre of Polymer Systems, University Institute, Tomas Bata University in Zlin, Tř. T. Bati 5678, Zlin, 760 01 Czech Republic

**Keywords:** Biomineralization, Swelling, Stimulus, Simulated biological solutions, Equilibrium swelling ratio

## Abstract

**Abstract:**

Biomaterials having stimuli response are interesting in the biomedical field. This paper reports about *swelling response* and *internal* *structural* of biomineralized (CaCO_3_) polyvinylpyrrolidone (PVP) carboxymethylcellulose (CMC) hydrogel having various thicknesses (0.1–0.4 mm). Samples were tested in aqueous solution using temperature ranges from 10 to 40 °C; pH varies from 4 to 9, time 60 min. In addition, an experiment was conducted in the presence of simulated biological solutions (SBS): glucose (GS), physiological fluid (PS) and urea (US) at temperature 37 °C and pH 7.5 for 180 min. It is noticed that the maximum swelling ratio reached in 30–40 °C at pH 7 in aqueous solution. Among biological fluids, the swelling ratio shows: US > PS > GS at temperature 37 °C, pH 7.5, time 150 min. The equilibrium swelling ratio of the test sample in SBS and their non-reformative apparent structure confirm that biomineralized (CaCO_3_) PVP–CMC hydrogel can be acclaimed for medical application like bone tissue engineering.

**Graphical Abstract:**

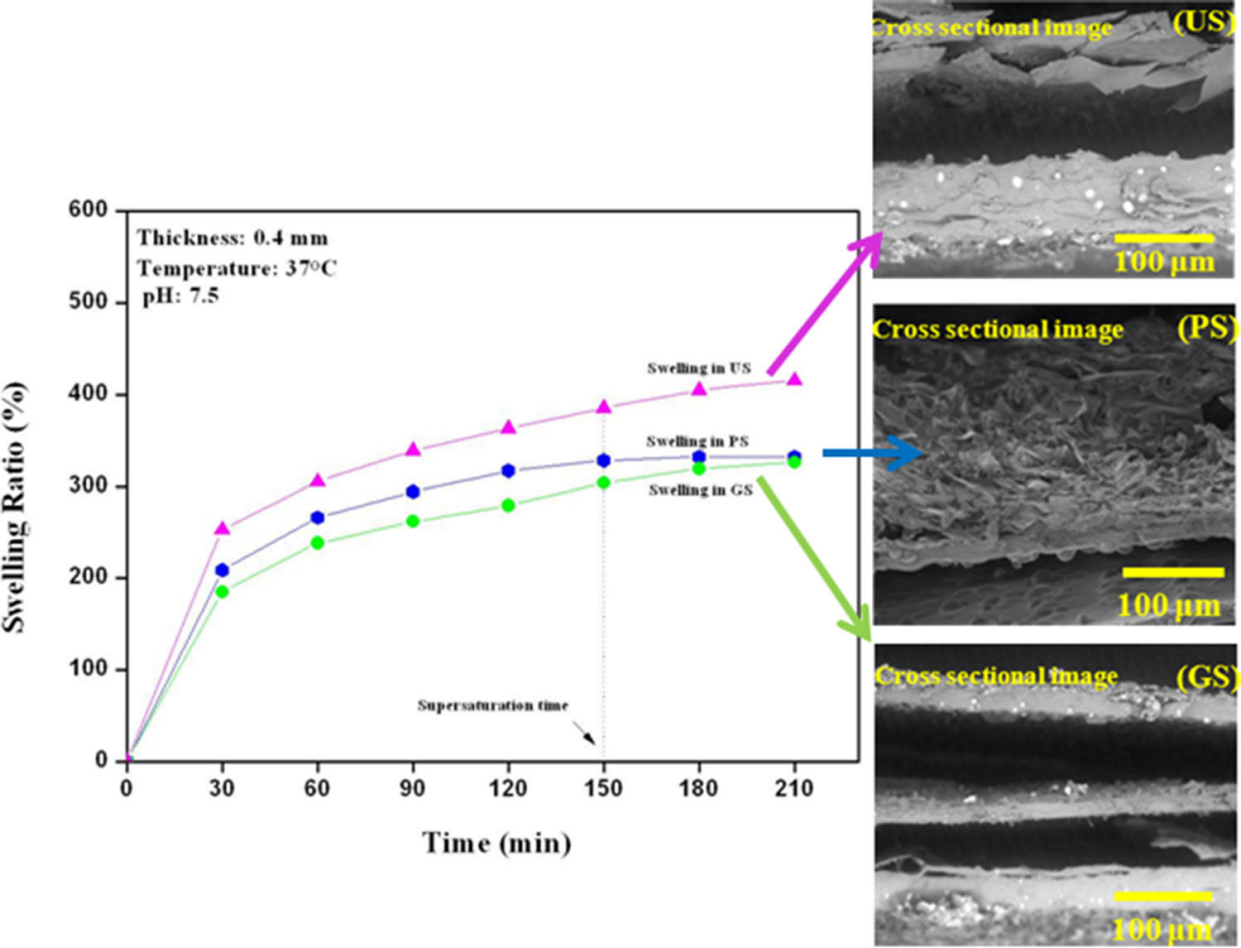

## Introduction

In the new era of biomedical field of tissue engineering and regenerative medicines, the utilization of materials generally comes in contact with any biological resources, in the form of either cells, tissues/organs, biomolecules, physiological fluids, etc., which desire the interdisciplinary scientific approach that merges the field of material science and technology, basic science and life science (Mano et al. 2007). In general, a tissue engineering process begins with the fabrication of a biologically compatible scaffold that will support the living cells for their attachment, proliferation and differentiation, and thus promotes tissue regeneration both in vitro and in vivo (Thavornyutikarn et al. 2014). Thus, during present days, intense interest has been given in the biomedical field of tissue engineering wherein several new biomaterials are constructs in the form of scaffold (i.e., polymeric material, bioceramics, biocomposites) can be implanted in patients to replace failing or malfunctioning organs (Mano et al. 2007). Moreover, several materials have been proposed to have their applications in biomedical fields among which significant attention has been given on the use of polymers, especially biopolymers and bioactive polymers. Polysaccharides and their derivatives have attracted much interest, especially the blends of natural and synthetic polymers are used because of their special properties in the form of soft rubbery nature resembling the living tissues, elasticity and also low cost (Zhu and Marchant 2011; Karadag et al. 2005). Among various kinds of polymeric systems that have been utilized till today, hydrogels have gained noticeable interest by the material scientists and are interpreted from different point of view (Kim and Park 2014; Sadeghi and Hosseinzadeh 2010).

Hydrogels are a unique class of three-dimensional macromolecular network structure that is highly suitable for several biomedical and tissue engineering applications because of the resemblance of their structure to the biological tissues (Tomic et al. 2010; Roy and De 2014). Moreover, these hydrogels have a network structure which indicates that cross-links should be formed to avoid dissolving it in the solution and thus maintaining the structural integrity within the aqueous phase (Kaith et al. 2010). Further the hydrogels being hydrophilic in nature, they have the capacity to imbibe and store huge water content within them, thus maintaining their 3D structure (Zhao et al. 2006; Tyliszczak 2011; Ismail et al. 2012). The hydrophobicity nature of the hydrogels is because of the presence of chemical moieties like hydroxylic (–OH), carboxylic (–COOH), amidic (–CONH–), sulphonic (–SO_3_H), etc. and also the other functional groups present in the backbone of the polymers (Ganji et al. 2010). Another aspect of the hydrogels is that the changes observed with the variations seen in surrounding environment of pH, temperature, simulated biological solutions, ionic strength, electric field stimulus and so on (Roy and De 2014; Rodkate et al. 2015; Sadeghi and Koutchakzadeh 2007; Chang et al. 2010). Due to the presence of some functional groups within the background of the polymeric chain, hydrogels become sensitive to the environmental conditions (Nesrinne and Djamel 2013). Hydrogels can undergo swelling from ionic network when pH, temperature or any other stimulus become dependent. Here, the high swelling efficiency of hydrogels is concerned with the electrostatic repulsion within the ionic groups present in the polymers and also the osmotic pressure inside and outside the hydrogel (Roy and De 2014). This type of study becomes interesting in the scientific area to be utilized in the advanced technologies. These types of hydrogels are referred to as stimuli responsive and smart hydrogels (Rodkate et al. 2015). Till now, these hydrogels with unique characteristics have been implemented and given importance in several field of biomedical applications: wound healing, cell encapsulation, drug delivery, tissue engineering, etc. (Rodkate et al. 2015; Nesrinne and Djamel 2013). Further, uses of hydrogels have also been noticed in carrying out the biomineralization process in several in vivo environments (Kim and Park 2014).

Nowadays, researchers related to bioinspired and biomimetic biomineralization for the preparation of user friendly and cost effective functional biomaterial are giving much emphasis. Material scientists are focusing much on the use of polymeric-based matrices as a promising tool for the development, repair and regenerate the functional tissues, organs, etc. of the body (Rauch et al. 2012). In material science, mimicking the natural structure design offers the great advantage in developing hybrid materials that combine the properties of organic polymers and inorganic phase. The combination of organic–inorganic phase leads to strengthening the properties of the prepared material (Rauch et al. 2012). Numerous studies have been proposed till now on bioinspired matrix-based biomineralization. Mineralization carried out on the matrix, which are fibrous and porous in nature, can provide better framed structure on which growth and nucleation process of inorganic crystals can occur (Kim and Park 2014). However, to accomplish this process, several biominerals play an important role as they possess specific shape and properties. Further, most of the studies carried out till now are focused on the development of crystal formation on gels, fibers, or film (Kim and Park 2014). Also, it is interesting to understand the role of chemical motifs that are mediated by the matrix properties in the form of its pore size, its composition, functionalization with active groups present within the polymeric matrix and cross-linking density (Nindiyasari et al. 2015). Biominerals in the form of hydroxyapatite, calcium phosphate/carbonate are used to carry out the process of biomineralization, thus forming organic–inorganic hybrid materials (Rauch et al. 2012; Feng-ju et al. 2012). Among all the biominerals, calcium carbonate (CaCO_3_ as calcite) is one of the most abundant minerals found in the nature produced by organisms. It has also several industrial applications like fillers in paints, plastics, or paper (Feng-ju et al. 2012). From the literature survey, it was found that mostly calcite crystal shows the crystallographic orientation within the hard tissues (Nindiyasari et al. 2015). Further, the formation of crystals within the matrix could be explained by the classical theory of crystallization which can be elaborately explained by the mesocystal theory (Rauch et al. 2012). Several studies have revealed the formation of crystals in various forms when comes in contact with the organic gel like matrixes.

In this aspect, it is already reported about the use of different kinds of matrixes which are mainly prepared with natural polysaccharides, i.e., alginate, cellulose, chitosan, gelatin, collagen, guar gum, dextran, etc. (Chunyu et al. 2011; Giridhar and Akanksha 2011) or peptoid nanosheets (Jun et al. 2015). Among all these, in our study cellulose (in derivative form) had been chosen as the best candidate to form hydrogel as cellulose is abundantly available in nature, biodegradable, having good water absorbing capacity and biocompatible too (Sadeghi and Hosseinzadeh 2010; Rodkate et al. 2015). In due course, to accomplish and maintain both hydrophilic and mechanical properties within the so-called “biobased hydrogel”, PVP is added to form a blend of carboxymethylcellulose (CMC) and polyvinylpyrrolidone (PVP) within the hydrogel. The interesting properties of this PVP–CMC hydrogel have already been reported by the researchers of Tomas Bata University in Zlin (Roy et al. 2010, 2012; Saha et al. 2010, 2011). Because of adventitious properties of PVP–CMC hydrogel like: porous internal morphology, quite a good moisture/solvent/solution absorption capacity, flexible in nature for the preparation of sample for testing in different shape, size and thickness, this bio-based hydrogel has been chosen to be used as a matrix for the preparation of calcite (CaCO_3_) incorporated hydrogel. Finally, this calcite-filled hydrogel termed as biomineralized (CaCO_3_) PVP–CMC hydrogel which has been prepared following the liquid diffusion method (Rauch et al. 2012; Saha et al. 2013a, b; Shah et al. 2014, 2015a, b, c).

Swelling is one of the important properties of hydrogel, thus it is essential to investigate its absorption behavior in the presence of aqueous solution/fluid. Water absorption occurred due to the combination mechanism of hydration, dissolution and thermodynamically expansion of the macromolecular chain restricted by crosslinkages of hydrogel like material (Ismail et al. 2012). Here, it is essential to mention that (to the best of our knowledge) till now no other group has been reported about the swelling behavior of biomineralized (CaCO_3_) PVP–CMC hydrogel (Saha et al. 2013a, b, Shah et al. 2014, 2015a, b, c). Further, through study of swelling nature/features of this biomaterial was conducted in the presence of various stimuli: in different aqueous pH, different aqueous temperature and simulated biological solutions [SBS: glucose solution (GS), urea solution (US) and physiological solution (PS)]. Apart from this, considering the practical application of this biomaterial, investigation has also been conducted to see the influence of individual stimuli (temperature, pH and SBS: GS, US and PS) on the structural properties of the biomineralized (CaCO_3_) PVP–CMC hydrogel. In this paper, emphasis has been given to understand and elucidate about changes in terms of *swelling* and *structure* of the said biomaterial in the presence of intracellular fluid or cytosol (liquid found inside cells), temperature: 37 °C and pH: 7.5. This information is important to evaluate the response of bone marrow-derived human mesenchymal stem cells in terms of cell proliferation and differentiation to the osteoblastic phenotype (which is responsible for bone formation) and to design the subsequent experiments.

## Experiment

### Chemicals

PVP K30 (PVP: molecular weight 40,000), polyethylene glycol 3000 (PEG: average molecular weight 2700–3300) and agar were supplied by Fluka, Switzerland; carboxymethyl cellulose (CMC) was purchased from Sinopharm Chemical Reagent Co-Ltd (SCRC), China; glycerin was obtained from Lachema, Czech Republic; calcium chloride (CaCl_2_: molecular weight 110.99 g/mol, 97.0 %), Penta, Czech Republic; sodium carbonate-10-hydrate (Na_2_CO_3_: molecular weight 286.14 g/mol) was obtained from Sigma-Aldrich; d-Glucose was purchased from Lukes, Czech Republic, NaCl and Urea from Penta, Czech Republic.

### Preparation of biomineralized (CaCO_3_) PVP–CMC hydrogel

**Fig. 1:**
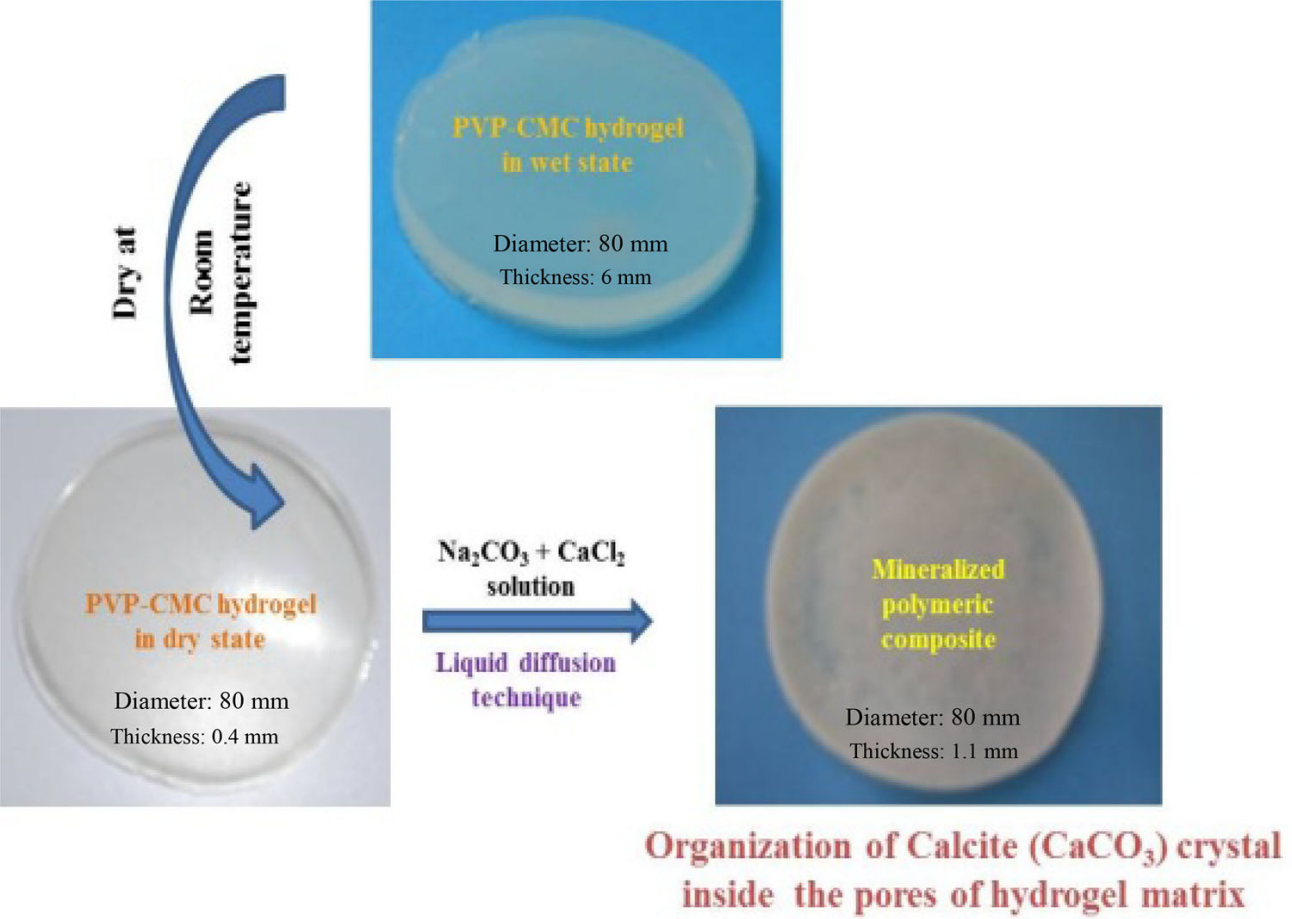
The physical appearance (*optical view*) of the biomineralized (CaCO_3_) PVP–CMC hydrogel before and after mineralization

Biomineralized (CaCO_3_) PVP–CMC hydrogel was prepared using a three-dimensional, crosslinked and porous matrix termed as “PVP–CMC hydrogel”. This hydrogel was prepared following the solution casting technique and implemented only physical cross-linking agent (i.e., moist heat and pressure) to achieve soft, white PVP–CMC hydrogel. The polymer solution (20/30/40/50 ml) was poured into a Petri dish (diameter: 80 mm) and kept at room temperature for gel formation. Thereafter, the freshly prepared PVP–CMC hydrogel was allowed to dry at room temperature (25–26 °C). The dry “PVP–CMC hydrogel” was utilized to prepare calcite-filled biomaterial. To achieve a biomineralized hydrogel, the simple liquid diffusion technique was adopted where the dried PVP–CMC hydrogel was immersed in the ionic solutions of 1 M Na_2_CO_3_ and 1 M CaCl_2_ simultaneously for 90 min. The obtained final form of biomaterial termed as “biomineralized (CaCO_3_) PVP–CMC” hydrogel and placed overnight for air drying. The dried samples were used for further investigations (i.e., swelling study). The physical appearance of the biomineralized (CaCO_3_) PVP–CMC hydrogel and their characteristics are presented in Fig. 1 and Table 1 respectively.

**Table 1:** Physical characteristics of biomineralized (CaCO_3_) PVP–CMC hydrogel

Initial polymer solution	PVP CMC Hydrogel						Biomineralized (CaCO_3_)PVP CMC Hydrogel					
	Before dry			After dry			Before dry			After dry		
Volume (ml)	Thickness (mm)	Weight (gm)		Thickness (mm)	Weight (gm)		Thickness (mm)	Weight (gm)		Thickness (mm)	Weight (gm)	
20	4.10–4.15	19–20	White, soft and fragile Size (diameter: 80 mm)	0.10–0.15	1.00–1.03	Off–white, transparent smooth and flexible Size (diameter: 80 mm)	0.40–0.45	6.60–6.70	Ivory, opaque, rubbery and flexible Size (diameter: 80 mm)	0.11–0.15	1.10–1.30	Off–white, opaque, coarse and Brittle, Size (diameter: 80 mm)
30	4.90–4.95	24–25		0.21–0.25	1.40–1.50		0.55–0.65	7.50–7.60		0.21–0.26	1.50–1.80	
40	5.90–5.95	32–35		0.30–0.35	2.00–2.01		0.80–0.90	9.70–9.80		0.31–0.36	2.40–2.70	
50	6.40–6.50	42–45		0.40–0.45	2.15–2.5		1.00–1.15	9.90–9.95		0.40–0.45	2.75–3.00	

### Swelling studies

The swelling study was performed using a dry sample (diameter: 25 mm × 25 mm and thickness: 0.1–0.4 mm) of biomineralized (CaCO_3_) PVP–CMC hydrogel. Gravimetric technique has been used to evaluate the swelling performance of biomineralized PVP–CMC hydrogel. Further, the influence of reaction parameters such as variation of pH, temperature in aqueous solution (water) and simulated biological solutions [i.e., glucose solution (GS), urea solution (US) and physiological solution (PS)] was investigated. The percentage of swelling ratio was determined using equation (Giridhar and Akanksha 2011; Fathi et al. 2013)

1 $$ {\text{Swelling}}\,{\text{Ratio}}\,(\% ) = \left( {W_{s} - W_{d} /W_{d} } \right) \times 100 $$

### Swelling studies at different temperature of aqueous solution

A uniform sized (25 mm × 25 mm) biomineralized (CaCO_3_) PVP–CMC hydrogel samples with a diverse thickness (i.e., 0.1, 0.2, 0.3 and 0.4 mm) were immersed in the 50 ml distilled water (aqueous solution, pH = 7) and then incubated in different temperature (i.e., 10, 20, 30 and 40 °C) for 60 min. Then, the weight of each set of swelled sample with different thickness was noted and their percentage of swelling at different temperature was calculated as per Eq. (1). Three replicas for each set of sample were prepared for estimation.

### Swelling studies at different pH of aqueous solution

Similarly, biomineralized (CaCO_3_) PVP–CMC hydrogel samples (size: 25 mm × 25 mm; thickness: 0.1, 0.2, 0.3 and 0.4 mm) were immersed in 50 ml solution of distilled water (aqueous solution having pH of 4.0, 5.0, 7.0, 8.0, and 9.0) and then incubated at room temperature (25–26 °C) for 60 min. The pH values were precisely noted using the pH meter from company Sension TM^+^. Finally, the percentage swelling at different pH was calculated as per Eq. (1). Three replicas for each set of sample were prepared for estimation.

### Swelling studies in simulated biological solutions

Glucose solution (GS, 5 g in 100 ml distilled water), urea solution (US, 5 g in 100 ml distilled water) and physiological solution (PS, 0.9 g in 100 ml distilled water) are considered as simulated biological solutions. Biomineralized (CaCO_3_) PVP–CMC hydrogel samples (size: 25 mm × 25 mm; thickness: 0.1, 0.2, 0.3 and 0.4 mm) were immersed individually in each simulated biological solution (pH: 7.5) and incubated at 37 °C. In each case, the observed physical changes (occurred due to swelling) were noted in every 30 min interval and the experiment was carried out until 180 min. The percentage swelling was calculated using same Eq. (1) for the case of simulated biological solutions as well. Three replicas for each set of sample were prepared for estimation.

### Instrumental analysis

For structural analysis of polymeric hydrogel scaffold (PVP–CMC–CaCO_3_) always lyophilized (swelled samples were freeze dried under –81 °C for 72 h and then lyophilized for 24 h to produce porous scaffold) samples were used. The morphology of swelled biomineralized (CaCO_3_) hydrogel in the presence of GS, US and PS was investigated using scanning electron microscopy (SEM: Phenom world Pro) which is operated in the high vacuum/secondary electron imaging mode at an accelerating voltage of 5–20 kV). All the images were taken at the magnification of 100×–10k× and always freeze drying samples were used for cross-sectional studies.

Fourier transform infrared spectroscopy (FTIR) was used to determine physical–chemical structure of the biomineralized (CaCO_3_) PVP–CMC hydrogel after swelling in GS, US and PS. The spectra were obtained at wave number of 2000–600 cm^−1^ at room temperature with uniform resolution of 2 cm^−1^. Attenuated total reflectance ATR-FTIR was used with NICOLET 320 FTIR Spectrophotometer with “Omnic” software package.

## Results and discussion

### Swelling behavior of biomineralized (CaCO_3_) PVP–CMC hydrogel

Swelling capacity usually becomes prime significance in many practical applications of biomedical field that includes either personal hygiene products, drug delivery systems or in tissue engineering and also in agriculture as water releasing or storing system. This swelling capacity is said to be affected when comes in contact with external solutions like different ions, salt concentrations and valencies. The favorable property dealing with the hydrogel is the ability to swell when kept in any solvent. One of the best theories that explains the swelling mechanisms is the Donnan equilibrium theory which states that there always exist electrostatic interactions and osmotic pressure between inside and outside of the gel (Sadeghi and Hosseinzadeh 2010). When any hydrogel comes in contact with the solvent molecules, the solvent tries to attack the hydrogel surface and penetrate within the polymeric network structure. The hydrogel possesses any of the acidic or basic pendant groups within its polymeric backbone structure. If the acidic group is present, then the H^+^ ions move out and combine with the OH^−^ ions and form H_2_O. In this way, charge neutrality gets balanced. If the cation concentration rises, then there is an increase in the osmotic pressure in the gel system which finally causes shrinkage in it. However, the equilibrium stage is achieved within the swelling phenomenon when the elastic force within the network structure of the gel as well as osmotic pressure outside balances (De et al. 2002). In this series of experiment, swelling studies of biomineralized (CaCO_3_) PVP–CMC hydrogel were performed using different stimulus like: temperature, pH, and several simulated biological solutions which has been explained below.

### Effect of temperature on swelling

**Fig. 2:**
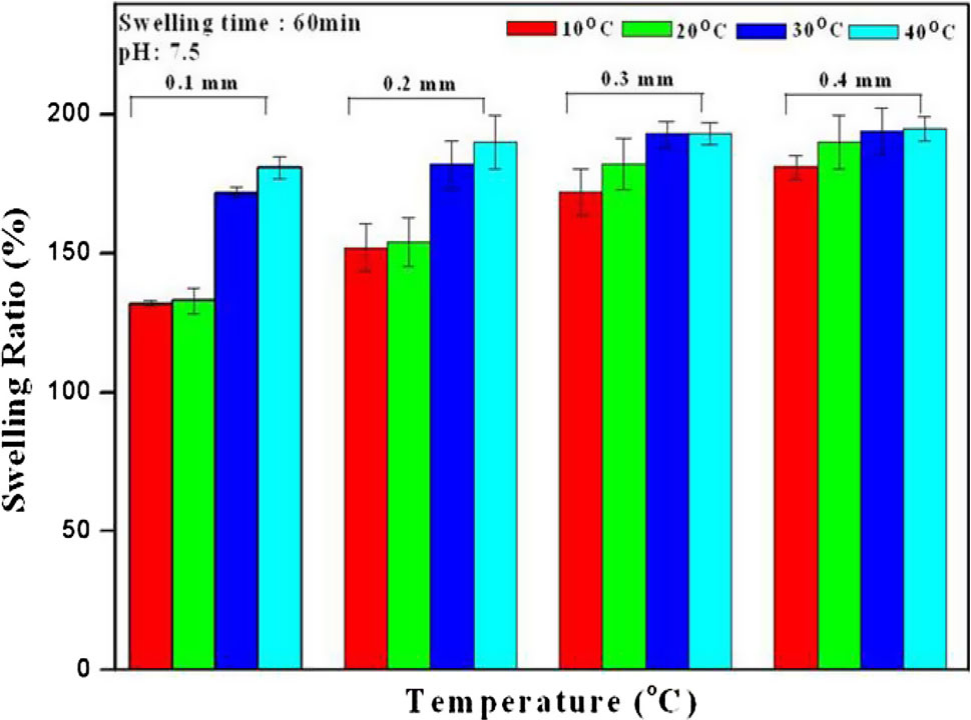
Effect of temperature on swelling of biomineralized (CaCO_3_) PVP–CMC hydrogel

The effect of temperature on swelling of biomineralized (CaCO_3_) PVP–CMC hydrogel is depicted in Fig. 2. It is noticed that irrespective of sample thickness (varies between 0.1 and 0.4 mm), the swelling capacity rises with increase of temperature, even though at lower temperature (10 or 20 °C), 0.1 and 0.2 mm thick samples showed lower range of swelling capacity. But, in the case of higher range of temperature, i.e., 30 and 40 °C, it shows more or less comparable range of swelling ratio similar to thick (i.e., 0.3 and 0.4 mm) samples. The reason behind the increase of swelling ratio of PVP–CMC–CaCO_3_ hydrogel with rise in temperature from 10 to 40 °C is basically due to the nature of internal polymeric interactions as well as the elasticity nature within the matrix. Moreover, at higher temperature the chain mobility increases which facilitates the network expansion and leads to increase in the ratio of swelling capacity (Gupta and Shivakumar 2012).

### Effect of pH on swelling

**Fig. 3:**
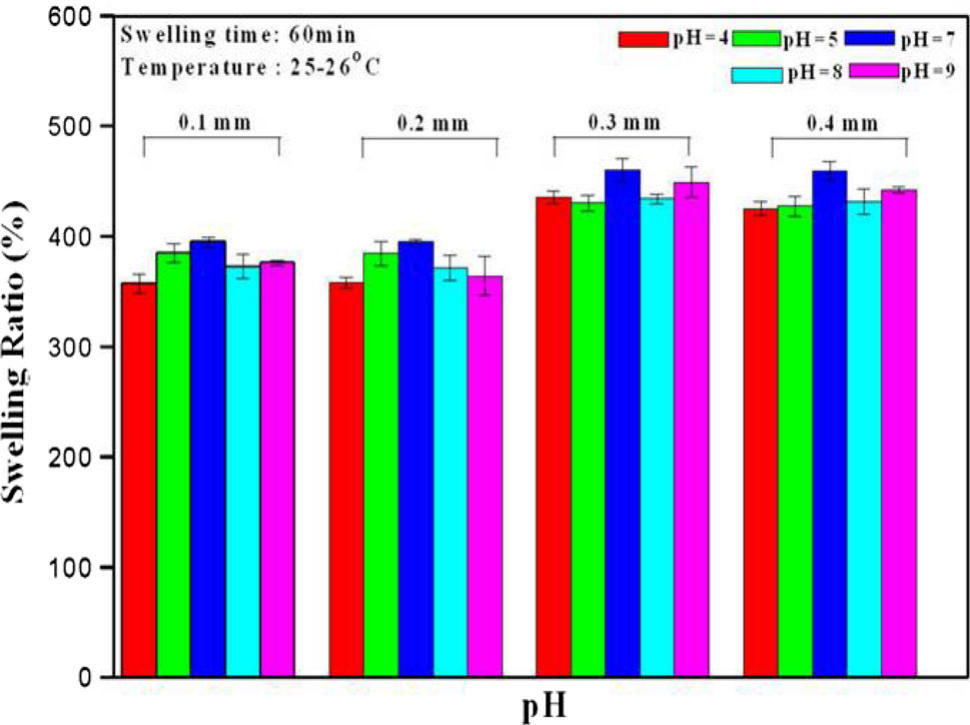
Effect of pH on swelling of biomineralized (CaCO_3_) PVP–CMC hydrogel

The effect of pH on swelling of the biomineralized (CaCO_3_) PVP–CMC hydrogel was investigated in acidic (4–5), neutral (7) and basic (8–9) range of pH and the results are shown in Fig. 3. Slight change in swelling ratio of the biomineralized hydrogels has been noticed when placed in various pH of the aqueous solution. It can also be noticed that irrespective of sample thickness at neutral pH = 7, all samples show a higher value of swelling ratio. This could be due to the presence of carboxylate (COO–) and amide groups in cellulose and PVP polymers. In pH = 7, water molecules undergo H_2_ bonding so generates more space for the molecules to penetrate inside, thus swells more. But, there was not much difference noticed when the biomineralized (CaCO_3_) PVP–CMC hydrogel is kept in the acidic and basic pH. Also, with the increase in pH above 7, the rate of swelling is lowered as there is dissociation observed in COOH– group present is cellulose, which gradually increases the movement of mobile ions that reduces the osmotic pressure. However, a little fluctuation has been noted in the swelling ratio with the increase in sample thickness. This indicates that change in the thickness of the samples slightly alters the absorbing capacity of the material (Kaith et al. 2010).

### Effect of simulated biological solutions (SBS) on swelling

**Fig. 4:**
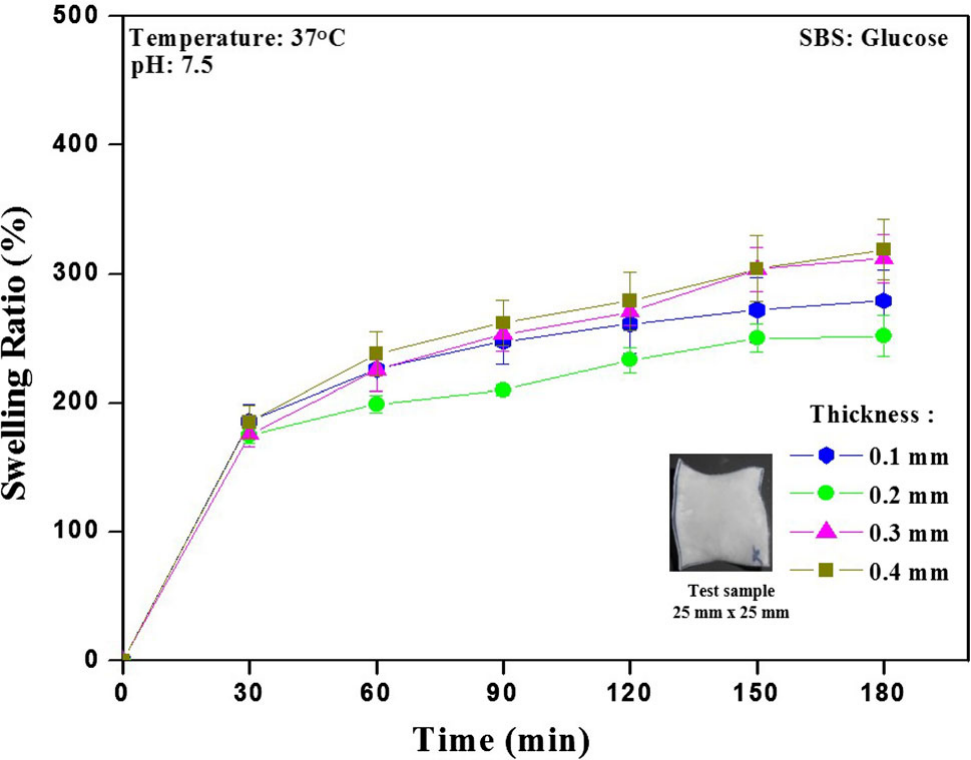
Swelling behavior of biomineralized (CaCO_3_) PVP–CMC hydrogel in glucose solution (GS)

**Fig. 5:**
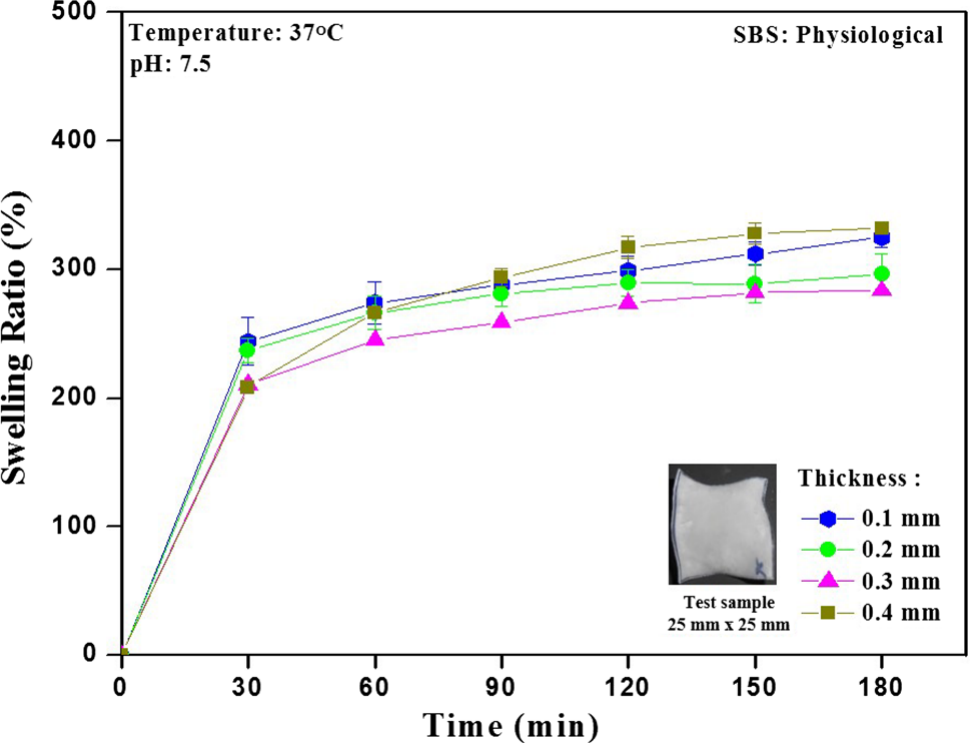
Swelling behavior of biomineralized (CaCO_3_) PVP–CMC hydrogel in physiological solution (PS)

**Fig. 6:**
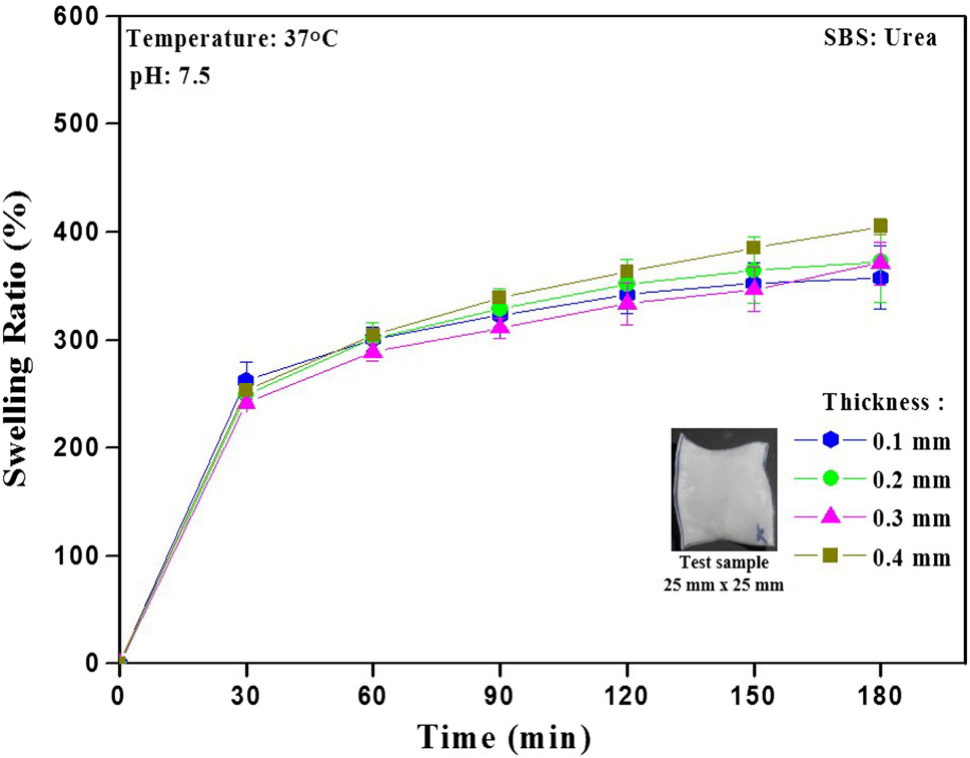
Swelling behavior of biomineralized (CaCO_3_) PVP–CMC hydrogel in urea solution (US)

To broaden the application of the biomineralized (CaCO_3_) PVP–CMC hydrogel and to examine the influence of simulated biological fluids on its swelling ratio, the swelling study was performed until 180 min in the presence of three different biological fluids: GS, PS and US holding the same environmental conditions of pH (7.5) and temperature (37 °C). The purpose of this investigation, i.e., exposure of the biomineralized (CaCO_3_) PVP–CMC in simulated biological fluids or simulated biological solution, is to understand the biomaterial condition when it comes in contact with biological/body fluid considering its application as a scaffold for bone tissue engineering or drug delivery. It is understandable from the literature that generally in the case of most healthy people, the body fluid maintains pH 7.5, temperature 37 °C and sugar level maintains the normal range which is as good as similar to the physiological solution but in the case of diabetic patient, the sugar level of body fluid will be little higher than healthy people or in the case of hyperuricemia patient, uric acid level will be excess in the blood and so on. Thus, considering the status of body fluid (healthy and un-healthy people), the effect of SBS on swelling of biomineralized (CaCO_3_) PVP–CMC hydrogel has been conducted. The observed results are depicted in Figs. 4, 5, and 6, respectively. In all the three simulated biological fluids, biomineralized (CaCO_3_) PVP–CMC hydrogel exhibited interesting results with their swelling behavior which indicates that this calcite-filled biomaterial will be helpful for cell proliferation which is in connection with bone tissue engineering. It can be seen in Figs. 4, 5 and 6 that the swelling capacity of hydrogel is gradually increased with the increase in time, though the values of swelling ratio vary for GS, PS and US. The reaction between a variety of fluids and composition of the test sample of biomaterial has great influences concerning uptake and swelling behavior.

Figure 4 represents the swelling trend of the biomineralized (CaCO_3_) PVP–CMC hydrogel in the presence of GS. Here, it can be revealed that the binding of glucose moieties with the mineralized hydrogels gradually increases the charge density within the hydrogels, hence enhances the hydrophilicity within the hydrogel which finally leads to the rise in the swelling capacity (Kim et al. 2014).

Figure 5 represents the swelling trend of the biomineralized (CaCO_3_) PVP–CMC hydrogel in the presence of PS. Like GS, in the physiological solution (0.9 % NaCl), there is a continuous uptake of PS which has been noticed by the hydrogel, and after certain time interval the absorption becomes stable. Here, during swelling studies, PS needs to overcome the osmotic pressure within the gel, and as osmotic pressure becomes lower water permeates inside the gels fast which leads to expansion of the gel (Sadeghi and Hosseinzadeh 2010).

Like GS and PS, the same trend of swelling behavior is observed in US which is depicted in Fig. 6. In urea solution, there is the presence of more hydrophilic sites like NH_2_^+^ and C=O and also urea being weak base can interact readily with COOH group of cellulose present in the hydrogel. Therefore, when urea reacts with water, it gains more hydrophilicity (Karadag et al. 2005; Kundakci et al. 2011). Thus, an increase in the hydrophilic groups within the urea aqueous solution will ultimately increase the swelling of biomineralized (CaCO_3_) PVP–CMC hydrogel.

**Fig. 7:**
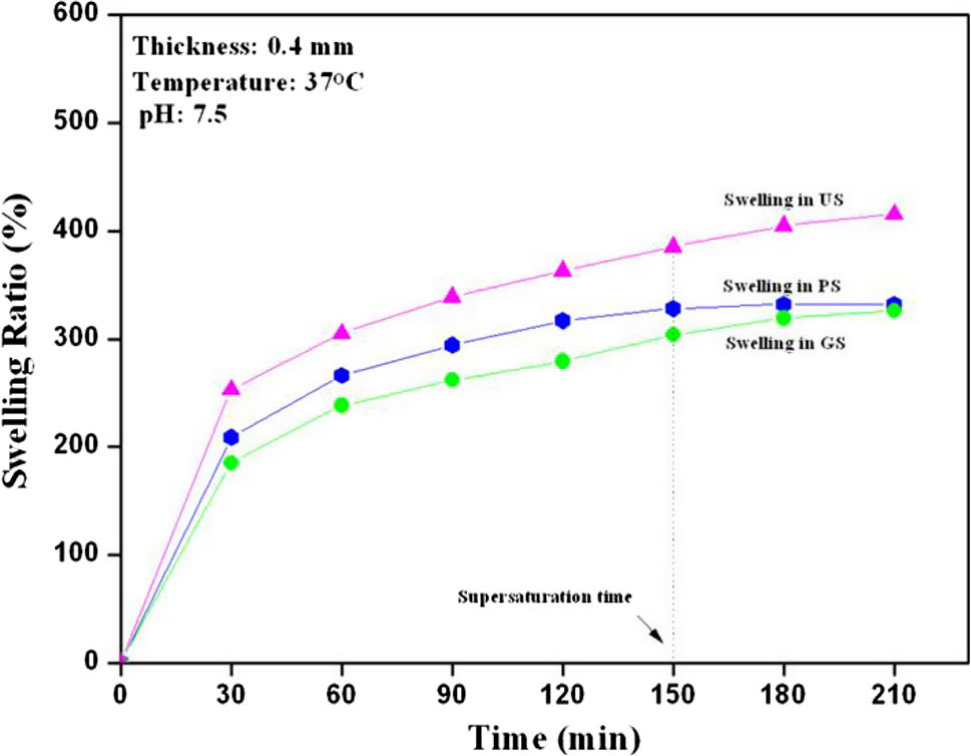
Swelling behavior and super saturation time of biomineralized (CaCO_3_) PVP–CMC hydrogel in simulated biological solutions

Further, the super-saturation swelling time (data represented in Fig. 7) has been identified from the swelling period (0–210 min) of biomineralized (CaCO_3_) PVP–CMC hydrogel when conducted the swelling study in the presence of GS, US and PS. It can be perceived from the Fig. 7 that the super saturation is attained in the time period of 150 min. After this, the stability in the swelling ratio has been noticed. It is well known that the swelling of any hydrogel is governed by the electrostatic repulsion of the ionic charges within the polymeric network structure. The more the hydrophilic groups present in the matrix, the more will be the swelling capacity. As far as the swelling behavior is concerned, two main important phenomenons are focused: (1) Donnan osmotic pressure and (2) elastic property within the polymeric network structure. When these two phenomena become equal, no further uptake of the solution takes place by the material and hence super-saturation stage is attained (Kim et al. 2014).

**Fig. 8:**
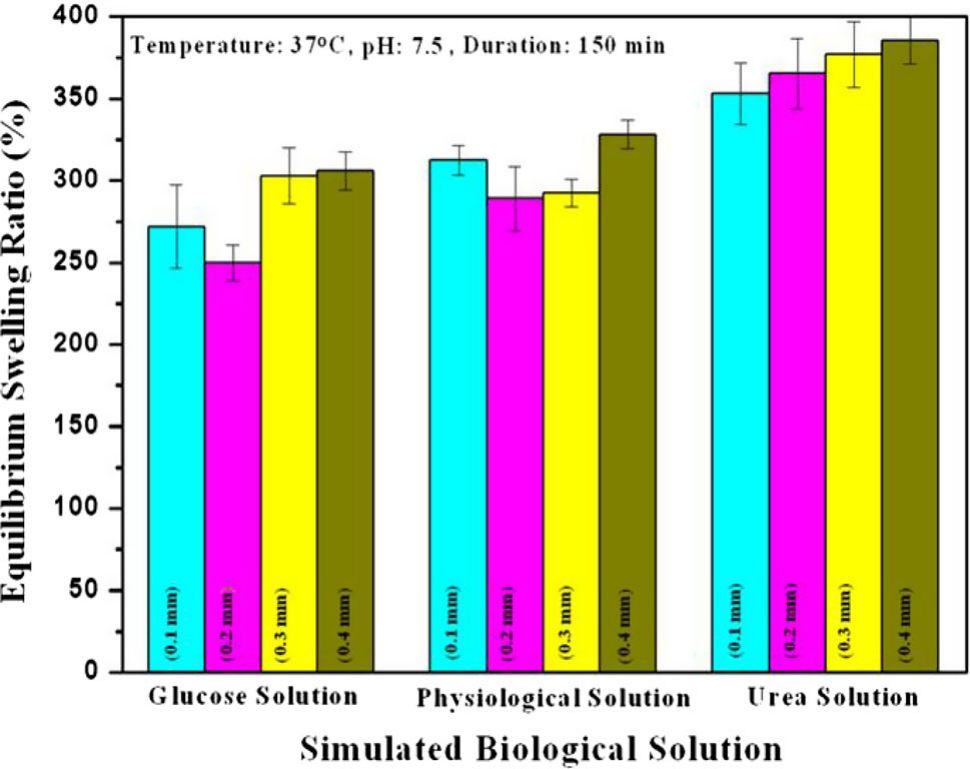
Equilibrium swelling ratio of biomineralized (CaCO_3_) PVP–CMC hydrogel in simulated biological solutions

The super-saturation point of the biomineralized (CaCO_3_) PVP–CMC hydrogel (thickness = 0.4 mm) with respect to the test sample of biological solutions (GS, PS, US, etc. which is usually present in cytosol/intracellular fluid) is shown in Fig. 8. Finally, at super-saturation point, the value of the equilibrium swelling ratio of biomineralized (CaCO_3_) PVP–CMC hydrogel (thickness: 0.1–0.4 mm) has been identified in the presence of simulated biological solutions (SBS: GS, PS and US). The values of equilibrium swelling ratio in terms of thickness are depicted in Fig. 8. The order of the swelling ratio obtained is as follows: US > PS > GS irrespective of sample thickness. The observed variation in the equilibrium swelling ratio may be due to the presence of non-uniform porous internal morphology of the biomineralized matrix/non identical filler (CaCO_3_) content inside the calcite-filled biomaterial. The equilibrium swelling value of biomineralized (CaCO_3_) PVP–CMC hydrogel is higher in US compared to PS and GS. The overall results obtained through this study indicated that there exists a strong electrostatic interaction within the biomineralized (CaCO_3_) PVP–CMC hydrogel. Moreover, apparently no deformation observed within the biomaterial due to the occurrence of swelling phenomenon (except increase of thickness).

### Morphology of biomineralized (CaCO_3_) PVP–CMC hydrogel (after swelling)

#### Effect of temperature

**Fig. 9:**
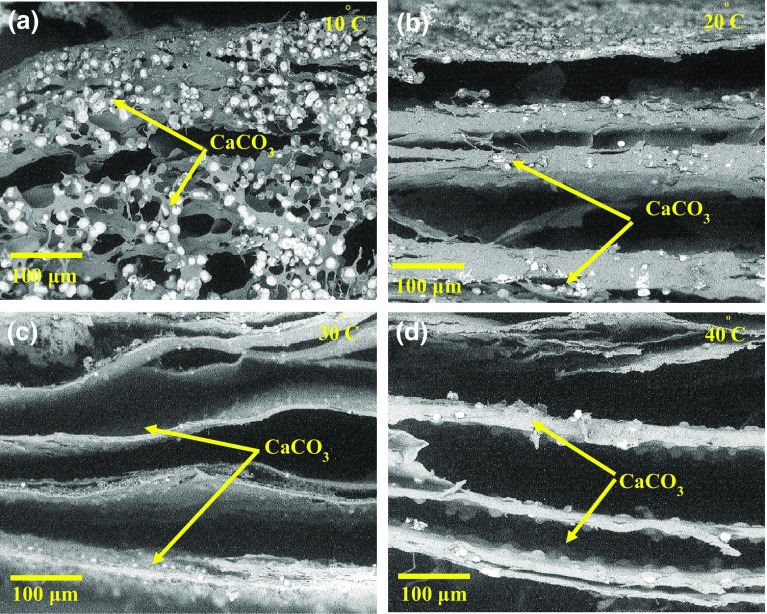
SEM Images of biomineralized (CaCO_3_) PVP–CMC hydrogel (thickness: 0.4 mm). Swelled in different temperatures: **a** 10 °C, **b** 20 °C, **c** 30 °C, **d** 40 °C

**Fig. 10:**
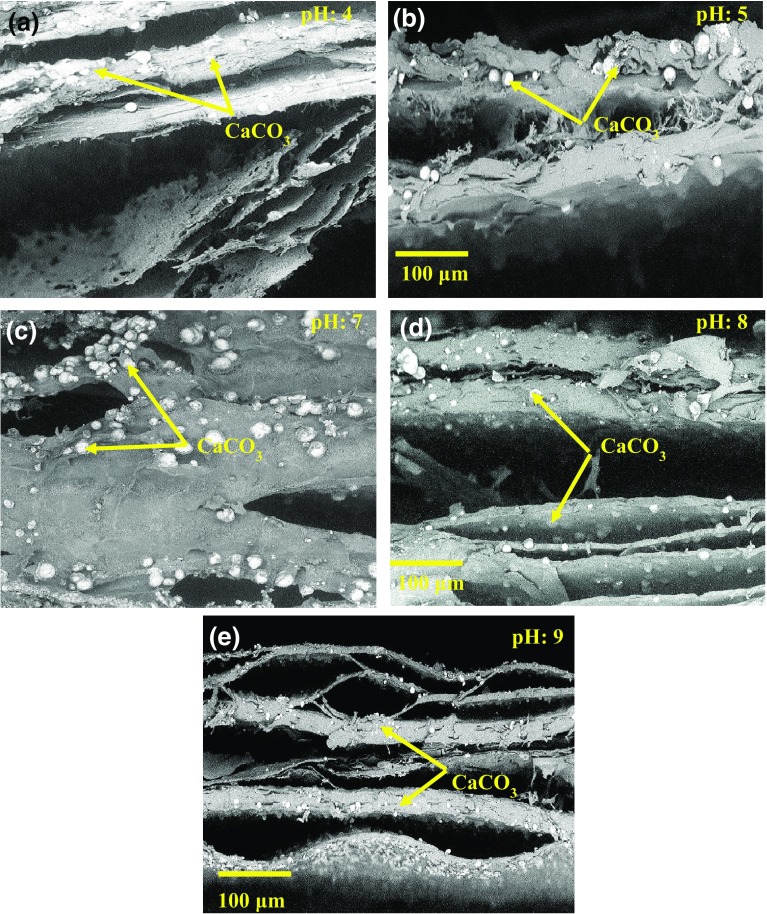
SEM Images of biomineralized (CaCO_3_) PVP–CMC hydrogel (thickness: 0.4 mm) swelled in different pH: **a** pH = 4, **b** pH = 5, **c** pH = 7, **d** pH = 8, **e** pH = 9

SEM generally provides the information about internal morphology, pore size and homogeneity/heterogeneity of the material. Figures 9 and 10 represent the SEM images of biomineralized (CaCO_3_) PVP–CMC hydrogel which are swelled in different temperatures (10, 20, 30 and 40 °C) and pH (4, 5, 7, 8, and 9). From the images of swelled hydrogels, one thing is commonly noticed that they exhibits discontinuous morphology with two phases, wherein polymer phase gets separated by the presence of internal unequal spaces between them (Ceylan et al. 2006). Moreover in all the figures, it is clearly visible the difference about the formation of flakes like structures due to swelling in which the crystals of CaCO_3_ are already embedded within the PVP–CMC hydrogel. Even though the thickness (i.e., 0.4 mm) of the biomineralized hydrogel sample is same, the increase in the temperature not only enhances the swelling ratio but also changes the internal structure of the biomineralized hydrogel.

#### Effect of pH

Like temperature, pH also exhibited the difference in internal morphology of the biomineralized (CaCO_3_) PVP–CMC hydrogel. It can be seen from the Fig. 10 that pH plays a significant role during swelling as well as the internal morphology of the biomineralized, which is different from the effect of temperature. More fractured planes and honey comb-like structures are developed within the hydrogel matrix due to swelling. The CaCO_3_ crystals (as droplets) are embedded tightly inside the hydrogel matrix throughout the surface of the planed cross-sectional structure.

#### Effect of simulated biological solutions (SBS)

**Fig. 11:**
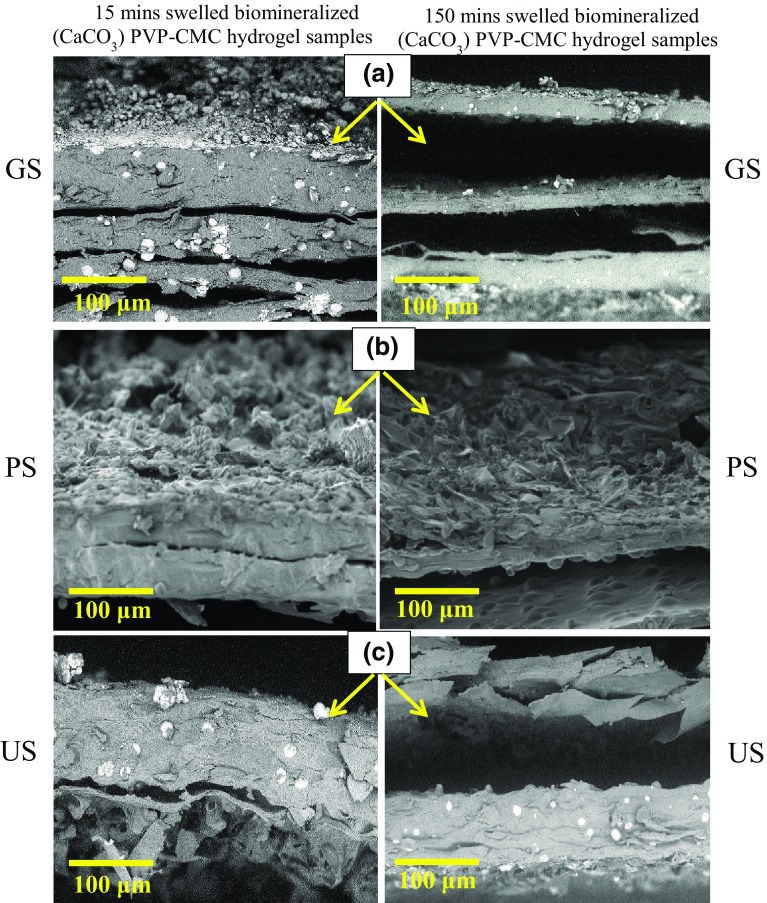
SEM Images of biomineralized (CaCO_3_) PVP–CMC hydrogel swelled in simulated biological solutions: **a** Glucose solution (GS), **b** physiological solution (PS) and **c** urea solution (US)

SEM micrographs (cross-sectional view) of the biomineralized (CaCO_3_) PVP–CMC hydrogel swelled in simulated biological solutions, i.e., GS, PS and US, are shown in Fig. 11. The results obtained are credited with the previously shown fact in case of swelling capacity wherein the presence of electrostatic interactions as well as more hydrophilic group increases the capacity of the sample to swell more. Therefore, in the SEM image of swelled biomineralized (CaCO_3_) PVP–CMC hydrogel, numerous bulked flakes-like structure with embedded calcium carbonate (CaCO_3_) is recognized in the case of GS and US except PS. Moreover, it can be seen from the Fig. 11 that not much difference is visible in terms of internal morphology with an increase in swelling time (between 15 and 150 min); however, the density increases within the hydrogel.

#### Physical and chemical nature of biomineralized (CaCO_3_) PVP–CMC hydrogel (after swelling)

**Fig. 12:**
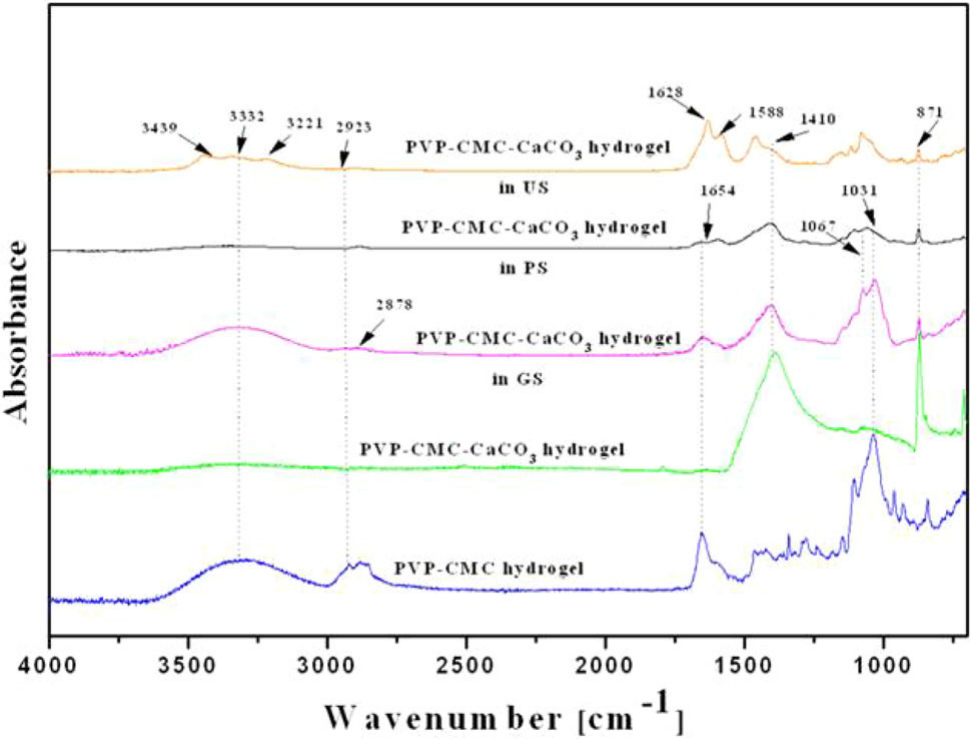
FTIR spectra of PVP–CMC, PVP–CMC–CaCO_3_ and swelled sample of PVP–CMC–CaCO_3_ in simulated biological solutions: GS, PS, US

Besides observed changes in swelling ratio in the presence of simulated biological solutions (GS, PS and US), the FTIR assay of biomineralized (CaCO_3_) PVP–CMC hydrogel sample (before and after swelling) has been performed to confirm the changes and shown in Fig. 12. The FTIR spectra of PVP–CMC hydrogel have been placed as a control. It can be seen from the figure that the strong absorption band of CO_3_^2−^ is present in all the peaks except pure PVP–CMC hydrogel with the range around 1410 and 871 cm^−1^ which confirms the deposition of calcite within the matrix (Shah et al. 2014; Ma et al. 2013). Further, it can be seen in all the spectra that a broad band of peak in the range of 3332 and 2923 cm^−1^ corresponds to hydrogen bonding forming –OH group of the polymer glycoside ring and the –OH stretching of water free and involved in hydrogel bonds. The peak showing around 1600 cm^−1^ corresponds to the amide I, amide II and free carboxylate groups of the CMC polymer. It is interesting to obtain the band at a range of 3439 and 3221 cm^−1^ in case of hydrogel when swelled in US. This peak corresponds to –N–H group of amide linkage which is developed due to the presence of urea in the swelled sample. It seems that during the swelling of PVP–CMC–CaCO_3_ hydrogel in the presence of US, no chemical reaction occurs between the existing –OH and –NH group after uptake of urea. Also, in the same urea swelled PVP–CMC–CaCO_3_ hydrogel, there are peaks seen at 1628 and 1588 cm^−1^, which are of amide –N–H and carbonyl group –C=O of urea. In the case of hydrogel samples swelled in the presence of GS, the peaks seen at 2878 cm^−1^ corresponds to the presence of –C-H group existing in its chemical structure. Further, in case of peaks in PS and GS, there is the presence of bands at a range of 1067 and 1031 cm^−1^ which are of –C–O– and –C–C– groups. All these observations confirming that even though the PVP–CMC hydrogel is filled with CaCO_3_, still the biomineralized sample has enough absorption capacity.

## Conclusions

In this work, a series of experiments concern with the swelling study of the biomineralized biomaterial (i.e., CaCO_3_–PVP–CMC hydrogel) was performed as “swelling nature of a material” is an important parameter of any biomaterials for biomedical or pharmaceutical application point of view. Different stimulus like temperature, pH, and simulated biological solutions like glucose (GS), physiological (PS) and urea (US) solution was used to investigate their response/changes in the form of swelling ratio and structure. The “physiological solution” was selected as its osmotic pressure is considered equivalent to human tissue fluids. “Urea solution” was chosen as urea is one of the important fluids produced in the body though kidney and also used in regeneration systems of artificial kidney machines wherein it is produced as toxic waste in the dialysate solution during the hemodialysis process of the patients. Furthermore, it is present in the blood in the amount of 6-20 milligrams per deciliter mg/dl as blood urea nitrogen (BUN) (Kamal 2014). Swelling test was also performed in the presence of “Glucose solution” because like urea, glucose is also present in the blood (an example of body fluid) less than 100 mg/dl (Dixon 2015). However, this level of glucose swings within the body throughout the day. In addition, it is important to mention that in all cases the swelling study of biomineralized (CaCO_3_) PVP–CMC hydrogel was performed at temperature 37 °C (as it is the normal temperature of internal body fluid) and pH 7.5 (as cell growing state pH remains little higher range than usual pH range, i.e., 7.0–7.4).

In conclusion, it can be mentioned that the biomineralized (CaCO_3_) PVP–CMC hydrogel is a smart biomaterial which showed an active response (i.e., change in swelling ratio) in all types of stimulus (i.e., Temperature, pH and SBS solutions). Certain changes in the internal structure of the biomaterial (during the swelling phenomenon) have also been observed. Moreover, through the entire experiment accomplished, there was no deformation noticed in the test material. As a whole, the aforementioned biomineralized (CaCO_3_) PVP–CMC hydrogel gives the fact that it has stimuli responsive behavior and can be recommended for its potential applications in several biomedical fields after performing the cytotoxicity and cell proliferation assay using several kinds of osteoblastic or stem cell, etc. which is in progress.
